# Characterizing efficient feature selection for single-cell expression analysis

**DOI:** 10.1093/bib/bbae317

**Published:** 2024-07-08

**Authors:** Juok Cho, Bukyung Baik, Hai C T Nguyen, Daeui Park, Dougu Nam

**Affiliations:** Department of Biomedical Engineering, Ulsan National Institute of Science and Technology (UNIST), 50, UNIST-gil, Ulsan 44919, Republic of Korea; Department of Biological Sciences, Ulsan National Institute of Science and Technology (UNIST), 50, UNIST-gil, Ulsan 44919, Republic of Korea; Department of Biological Sciences, Ulsan National Institute of Science and Technology (UNIST), 50, UNIST-gil, Ulsan 44919, Republic of Korea; Department of Predictive Toxicology, Korea Institute of Toxicology, 141, Gajeong-ro, Daejeon 34114, Republic of Korea; Department of Biological Sciences, Ulsan National Institute of Science and Technology (UNIST), 50, UNIST-gil, Ulsan 44919, Republic of Korea; Department of Mathematical Sciences, Ulsan National Institute of Science and Technology (UNIST), 50, UNIST-gil, Ulsan 44919, Republic of Korea

**Keywords:** single-cell RNA-sequencing, feature selection, clustering, trajectory analysis

## Abstract

Unsupervised feature selection is a critical step for efficient and accurate analysis of single-cell RNA-seq data. Previous benchmarks used two different criteria to compare feature selection methods: (i) proportion of ground-truth marker genes included in the selected features and (ii) accuracy of cell clustering using ground-truth cell types. Here, we systematically compare the performance of 11 feature selection methods for both criteria. We first demonstrate the discordance between these criteria and suggest using the latter. We then compare the distribution of selected genes in their means between feature selection methods. We show that lowly expressed genes exhibit seriously high coefficients of variation and are mostly excluded by high-performance methods. In particular, high-deviation- and high-expression-based methods outperform the widely used in Seurat package in clustering cells and data visualization. We further show they also enable a clear separation of the same cell type from different tissues as well as accurate estimation of cell trajectories.

## Introduction

Single-cell RNA sequencing (scRNA-seq) has enabled the investigation of the transcriptome of individual cells for various disease and cellular conditions [1–3]. scRNA-seq yields the expression profiles of thousands or higher number of cells that are typically sparse and noisy. A primary goal of scRNA-seq is the accurate identification of underlying cell types, which is performed by unsupervised clustering of cells and dimension reduction. For an efficient and accurate analysis, hundreds to thousands of features (genes) that are deemed to have different distributions between cell types are selected and used for downstream analysis. One of the most widely used feature selection methods has been the highly variable gene selection available in Seurat package, denoted as *SeuratVst* in this article [4], and other methods used deviation or dropouts to select features [5].

Previous benchmarks have shown that feature selection impacted the scRNA-seq data analysis [5–7]. However, two different criteria have been used to assess feature selection methods, lacking consistent recommendations. For example, Andrews and Hemberg assessed the proportion of ground-truth marker genes included in the features between seven feature selection methods, where the consensus of the methods showed a favorable performance [6]. However, other studies used the accuracy of cell clustering as assessed on the basis of ground-truth cell types, where high-deviation genes (HDG), high-expression genes (HEG) [8], and NBDrop, a dropout-based feature selection method [6] performed well [5, 9]. While these studies tested cell clustering for a number of datasets, they did not indicate why some feature selection methods perform better than others.

In this article, we compared the performance of 11 feature selection methods and demonstrated the discordance between the two criteria. We note that feature selection should be primarily aimed at accurate cell-level analysis such as cell clustering, as it subsequently enables the accurate analysis of genes (e.g. marker gene identification [10, 11] or ligand–receptor interaction between cell types [12, 13]). We performed simulation test to compare the proportion of ground-truth genes captured by each feature selection method, and then compared the accuracy of clustering and data visualization using the ground-truth cell types for both simulation and real scRNA-seq data. We avoided using the cell type labels obtained from clustering analysis with a specific feature selection method.

Then, we compared the distribution of selected genes in their means between feature selection methods to characterize high-performance methods. The widely used SeuratVst estimated the mean–variance trend of genes to select features that were more variably expressed than expected [4]. This approach was intended to allow most genes to have a similar chance to be selected irrespective of their mean expression levels, as related to the first criterion of feature selection. We show that lowly expressed genes have much greater coefficients of variation (CVs: standard deviation divided by mean) compared to highly expressed genes, suggesting their deleterious effect on downstream analysis when selected.

## Results

For two normalization methods (sctransform (ver. 2) [14] and log-normalization [4]), we tested 11 feature selection methods as follows: SeuratVst, HDG, HEG, two combinatorial methods (denoted as HDG$\mathbf{\cap}$SeuratVst and HEG$\mathbf{\cap}$SeuratVst) that used the features commonly selected between two methods, DUBStepR that leveraged gene–gene correlations [7], four dropout-based methods (NBDrop, NBDisp, M3Drop, and HIPPO) [6, 15] and dispersion-based method in Seurat (denoted as *SeuratDisp*) [4]. We used the community-based clustering algorithm provided in Seurat package [4]. Sctransform used filtering of very lowly expressed genes and lowly dispersed genes ($\mu <0.001,\kern0.5em {\sigma}^2\le \mu$), and such gene filtering was commonly applied to both normalization methods.

### Simulation test

**Figure 1:**
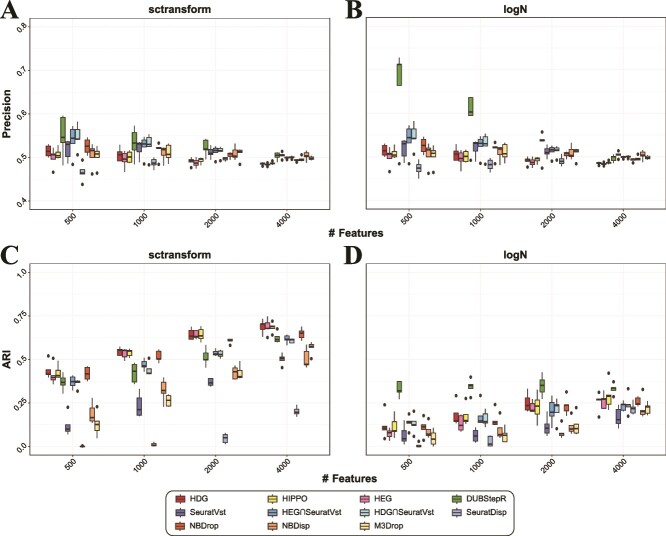
Comparison of 11 feature selection methods using two criteria: the proportion of ground-truth (differentially expressed) genes included in the features (precision) and the clustering accuracy as measured by ARI for ground-truth cell types. The results for five simulated datasets with overall zero rates 90% after gene filtering are shown. Precision and ARI of each method are compared for different numbers of high-scoring features 500, 1000, 2000, and 4000. The precision results are shown for (A) sctransform and (B) log-normalization. ARI results for (C) sctransform and (D) log-normalization.

We simulated scRNA-seq datasets with eight and five clusters (cell types) using splatter package [16] (See Methods). For the eight-cluster case, we tested two sparsity levels: 90% and 70% overall zero rates after gene filtering, each repeated five times (Fig. 1; Fig. S1). For all cases, SeuratVst included more ground-truth features (differentially expressed genes simulated in each cell type) than HDG and HEG (Fig. 1A and B; Fig. S1A and B). However, the opposite results were observed when the clustering accuracy was tested; the adjusted rand index (ARI) using SeuratVst was markedly lower than those using HDG and HEG (Fig. 1C and D; Fig. S1C and D). Interestingly, the combinatorial methods (HDG$\mathbf{\cap}$SeuratVst, HEG$\mathbf{\cap}$SeuratVst) were among the best in capturing the ground-truth genes, however, they exhibited only an intermediate clustering accuracy. HIPPO also showed a good clustering accuracy overall, and NBDrop showed a decent performance for both criteria. DUBStepR performed the best with log-normalization for both criteria, but it ranked fifth or lower in ARI with sctransform-normalization. However, DUBStepR exhibited the best ARIs for smaller feature numbers (500 and 1000) even with sctransform when less sparse data were used (Fig. S1), as this method leveraged gene correlations that were better estimated with less sparse data. We note that the overall ARI’s were higher with sctransform compared to those with log-normalization. These results demonstrate that the capability of feature selection to include many ground-truth genes does not necessarily yield accurate clustering results.

**Figure 2:**
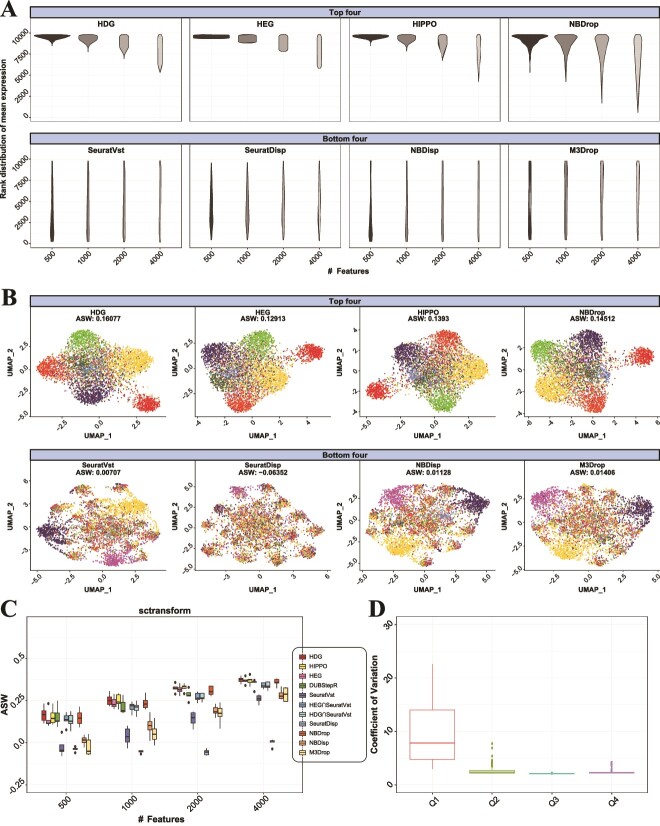
The distribution of selected features and UMAP visualization are compared between feature selection methods for simulated scRNA-seq data (eight cell types). Sctransform is used to normalize the data. (A) Rank distribution of gene expression (mean cpm) for selected features and (B) UMAP visualization (500 features) are compared between top four and bottom four methods in ARI along with corresponding ASW. The colors in B represent eight simulated cell types. (C) The visualization of data is compared using ASW between 11 feature selection methods for 500, 1000, 2000, and 4000 high-scoring features. (D) Coefficients of variation are compared between four quartile groups of gene expression (mean cpm). Q1 indicates bottom 25% lowly expressed genes, and Q4, top 25% highly expressed genes.

**Figure 3:**
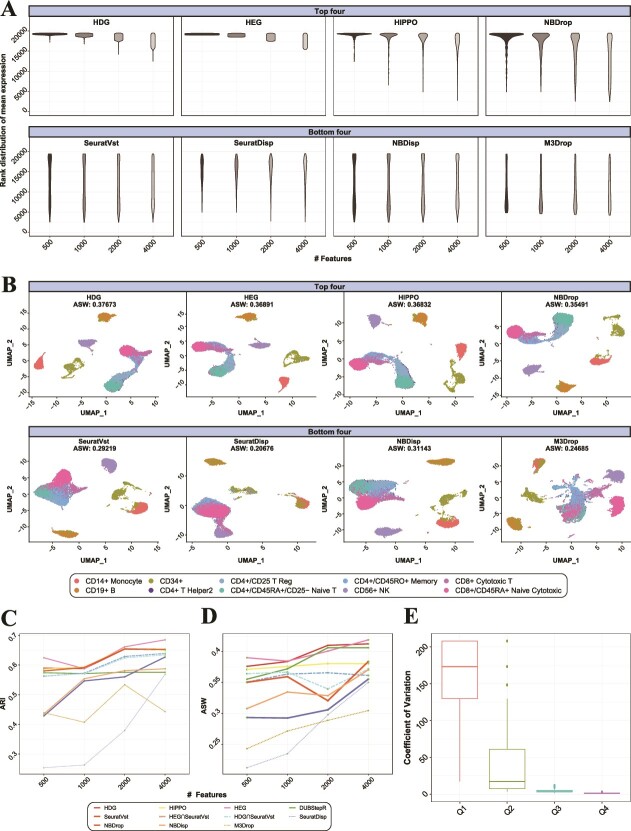
The distribution of selected features and UMAP visualization are compared between feature selection methods for FACS scRNA-seq data (10 cell types). Sctransform is used to normalize the data. (A) Rank distribution of mean expression (cpm) for selected features and (B) UMAP visualization (500 features) are compared between top four and bottom four methods in ARI. (C) The clustering accuracy and (D) visualization of data are overall compared between 11 feature selection methods using ARI and ASW, respectively. (E) Coefficients of variation are compared between four quartile groups of gene expression (mean cpm). Q1 indicates bottom 25% lowly expressed genes, and Q4, top 25% highly expressed genes.

Then, we compared the mean expression levels of genes captured by each feature selection method. Figure 2A and B; Figures S2A and B, S3A and B compared the rank distribution of selected features and cell-type visualization between the top four and bottom four methods in ARI. These methods were selected based on the test results for eight clusters, and one method (NBDisp) was replaced by HDG$\mathbf{\cap}$SeuratVst when five clusters were tested (Fig. S3A and B). Whereas the top four including HDG and HEG mostly selected highly expressed genes, the bottom four including SeuratVst tended to select genes evenly. We next compared the CV between genes using the S2N function in sctransform package [14]. Interestingly, the CVs for lowly expressed genes (bottom 25% mean values [Q1]) were on average more than four times higher than those for highly expressed genes (top 25% mean values [Q4]) (Fig. 2D). Such ratio was dramatically increased to 143 times when real scRNA-seq data were analyzed (Fig. 3E). This suggests that lowly expressed genes are dominated by technical noise rather than biological variability, thus, they can make only a minor (or negative) contribution to cell clustering. Indeed, when we simply excluded 20–70% lowly expressed genes, the clustering by using SeuratVst was considerably improved (Fig. S4). We then compared the visualization of ground-truth cell types for 500, 1000, 2000, and 4000 high-scoring features using average silhouette width (ASW); HDG and HEG exhibited a consistently better visualization of the cell types compared to SeuratVst (Fig. 2B and C; Figs S2B, S3B and D).

### Analysis of real scRNA-seq data

We analyzed two scRNA-seq datasets with known ground-truth cell groups based on fluorescence-activated cell sorting (FACS) as well as another scRNA-seq dataset annotated using marker gene expression. Abdelaal and colleagues used the 10 PBMC (peripheral blood mononuclear cells)-sorted populations each of which was downsampled to 2000 cells [17]. Thus, the corresponding scRNA-seq data comprised 20,000 cells for ten cell types (Table S2). For this dataset, HDG and HEG also exhibited substantially higher clustering accuracy compared to SeuratVst (Fig. 3C), whereas the combinatorial methods exhibited an intermediate performance. DUBStepR exhibited a nearly constant ARI due to its strong gene filtering (min.cell = 5%), which limited feature selection. The genes selected by each method were distributed similarly to the simulation cases (Fig. 3A). The 10 ground-truth cell types were visualized for each feature selection method (Fig. 3B and D; Fig. S5); HDG and HEG exhibited consistently better ASWs compared to SeuratVst. In particular, HDG and HEG distinguished CD8+ cytotoxic T cells from CD4+ T cells more clearly than SeuratVst (Fig. 3B). Moreover, HDG and HEG well separated CD4+ T cell lineage including CD4+/CD25+ T Reg, CD4+/CD45RA+/CD25− Naive T, CD4+/CD45RO+ Memory T cells, and CD4+ T helper2 on the 2D UMAP, while the bottom four methods did not clearly separate them. The distinct expression patterns between CD8+ and CD4+ T cell lineages in the HDG and HEG results indicated their distinct immune functionalities; T helper cells facilitate the B cell response, while T cytotoxic cells meditate cell killing [18]. Our results demonstrated that the high noise levels in lowly expressed features deteriorated the accuracy of both the clustering and dimension reduction, and HDG and HEG provided sufficient resolution to discern and visualize subtle cell types without using lowly expressed features.

We then tested HDG, HEG, and SeuratVst for discerning different sexes in the same cell type (endothelial cell) across different tissue origins of mice. Paik and colleagues [19] revealed organ-specific transcriptomic features and sex-related disparities in endothelial cells by analyzing scRNA-seq data from the Tabula Muris consortium [20]. From this data, we selected 3487 endothelial cells originating from four different organs, and each organ group included two subcategories, male and female, constituting eight cell groups in total. As the number of features used increased, ASW kept increasing in all three methods, and HDG and HEG consistently performed better than SeuratVst across different feature numbers (Fig. S6A). The endothelial cells were mainly clustered by their organs with minor differences between sexes in each organ. Interestingly, when HDG or HEG was used, clear separation between sexes was observed for brain and heart, whereas SeuratVst produced indistinguishable clusters between sexes (Fig. S6C). Then, we focused on discerning the two sexes within each organ; the ASWs in each organ were calculated and their average weighted by the cell counts in each organ was compared between feature selection methods. This test also showed a superiority of HDG and HEG over SeuraVst (Fig. S6B). Interestingly, HDG showed a dramatic increase in ASW as the number of features used increased, while HEG exhibited steady and high ASWs.

**Figure 4:**
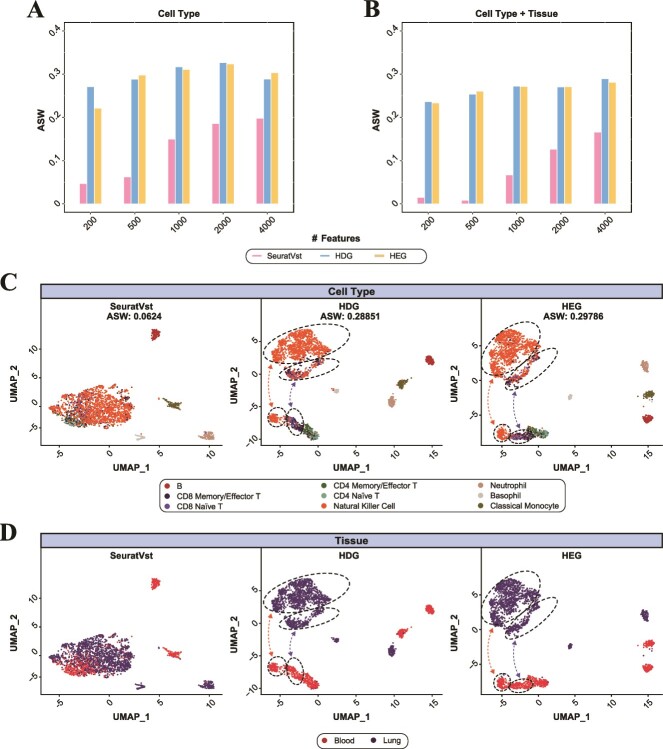
Comparisons of three feature selection methods (HDG, HEG, and SeuratVst) for classifying immune cell scRNA-seq data. Sctransform-normalization is used. (A) The nine cell types are annotated using singleR package and the ASW is compared. (B) Using the tissue information (blood and lung) added three more cell types and corresponding ASW for 12 types are compared. (C) UMAP visualization of nine cell types for 500 high-scoring features. Dashed ellipses indicate that three cell types (CD8 memory/effector T, CD8 Naïve T, and natural killer cell) are clearly separated between different tissues in HDG and HEG results. (D) The two tissue origins are shown (blood and lung).

Additionally, we tested whether HDG and HEG were capable of discerning detailed cell types of immune system on the basis of marker gene expression. Travaglini and colleagues comprehensively analyzed scRNA-seq data for lung and circulating blood samples comprising diverse cell types [21]. From the data, we selected 2318 immune cells that were matched to FACS bulk RNA-seq reference. These cells were annotated with nine cell types using singleR package [22] based on the FACS reference. By considering the tissue origin (lung and blood), these cell types were further divided into 12 cell types (Table S2). The visualization of cell types was compared between HDG, HEG, and SeuratVst with or without the tissue information (Fig. 4A and B). The ASW of SeuratVst kept increasing as the number of features used increased; however, HDG and HEG showed a steady and overall better performance compared to SeuratVst. In particular, the three cell types (CD8 Memory/Effector T, CD8 Naïve T, and Natural Killer Cell) were clearly separated between lung and blood when HDG or HEG was used, while they tended to be represented in a single cluster with SeuratVst (Fig. 4C and D). Such clustering patterns were consistently observed for higher number of features, 1000–4000 (Fig. S7). As a result, the ASWs for SeuratVst were considerably lowered when the 12 cell type labels with the tissue information were used, while those for HDG and HEG showed only a marginal decrease (Fig. 4A and B).

Overall, we demonstrated the capability of HDG and HEG to distinguish complex cellular conditions from different tissue, organ or sex origins and their potential for delineating shifting or differentiating cells across different tissues.

### Effect of imputation on clustering analysis

There have existed various efforts to estimate missing values (imputation) in scRNA-seq data [23]. Imputation methods have been built on different assumptions of data distribution and implemented using *ad hoc* parameters such as cluster number; thus, imputation methods did not always improve downstream analysis of scRNA-seq data and none of them performed the best for various test conditions [23]. Here, we tested imputation for clustering of PBMC-sorted data. The data were imputed using SAVER [24], SAVERX [25], and scVI [26] followed by normalization and clustering. The ARI scores for eight feature selection methods were compared (Fig. S8); here, three methods, NBDrop, NBDisp, and M3Drop were excluded because they did not use imputed data. Notably, SAVER improved ARIs for most feature selection methods and all three imputation methods improved ARIs for SeuratVst. This result showed recovery of biological signals and denoising by imputation might improve the clustering analysis through the respective feature selection methods. In addition, imputation tended to reduce the difference in ARI between feature selection methods and between the number of features used. We observed some positive effects of imputation on downstream analysis. However, imputation should be carefully used because it can introduce varying artifacts and their effects under different conditions (e.g., depth and sparsity) remain poorly understood.

### Effect of feature selection on trajectory analysis

**Figure 5:**
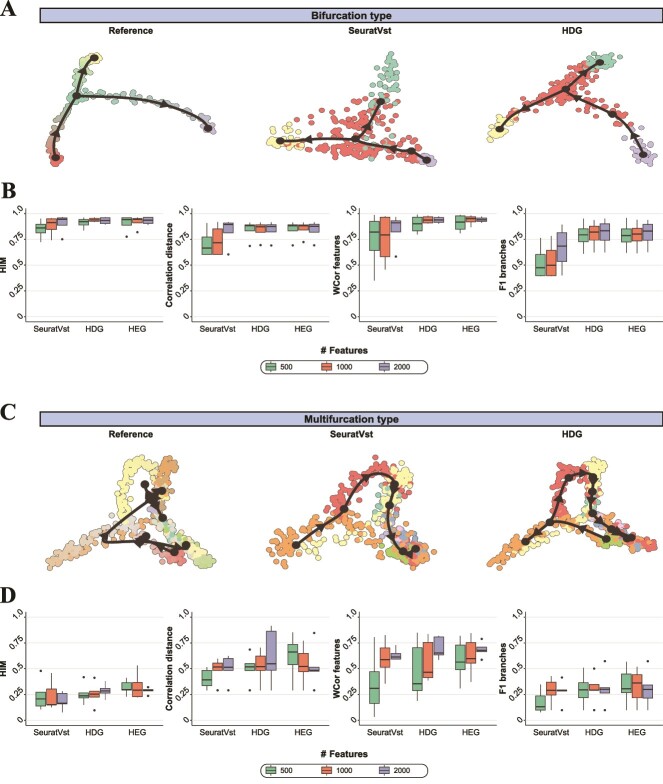
Comparison of trajectory inference (slingshot) between three feature selection methods (HDG, HEG, and SeuratVst). (A) Example of bifurcation trajectories for the ground-truth (reference), SeuratVst, and HDG. 500 features were selected from “synthetic_dyntoy_bifurcating_3” dataset using SeuratVst and HDG. (B) Four different accuracy metrics (HIM, correlation distance, WCor features, and F1 branches) were compared using four simulated bifurcation datasets between SeuratVst, HDG, and HEG for 2000, 1000, and 500 features. (C) Example of multifurcation trajectories for the ground-truth (reference), SeuratVst, and HDG. 500 features were selected from “synthetic_splatter_multifurcating_10” dataset using SeuratVst and HDG. (D) The accuracy metrics were compared using five simulated multifurcation datasets. In A and C, the black curves indicate the projected trajectory; the black dots indicate the milestones of a trajectory; the arrows indicate the direction of the trajectory progression.

Trajectory (aka, pseudotime) inference analyzes the order of cells along development or differentiation trajectories from scRNA-seq data [27]. This approach provided new opportunities for studying the cellular dynamics along linear, bifurcating, multifurcating, or tree-shaped trajectories. Saelen and colleagues systematically compared 45 trajectory analysis methods for accuracy, scalability, stability, and usability [27]. We used this benchmark to test the impact of feature selection on the trajectory inference. We used simulated datasets where the most exact trajectories were known and focused on the four bifurcation and five multifurcation datasets that included at least 500 cells and 3000 genes. All the tested datasets had been simulated based on real scRNA-seq data using the simulation packages, splatter [16], and dyntoy [28] (Table S3). We tested HDG, HEG, and SeuratVst methods for the trajectory inference using 500, 1000, and 2000 features. We tested Slingshot [29] that exhibited the best accuracy across various tests [27] and compared the four accuracy metrics between the feature selection methods (Fig. 5) as follows: the topology-based Hamming-Ipsen-Mikhailov metric (HIM), the quality of assignment of cells to branches/milestones (F1 branches), the cell positions (Correlation distance) [27], the accuracy of the differentially expressed features along the trajectory (WCor features). HDG and HEG overall provided favorable trajectory estimations for both bifurcation and multifurcation compared to those of SeuratVst. In the bifurcation cases (Fig. 5A and B), 500 HDGs closely estimated the reference trajectory, while 500 HVGs exhibited a rather dispersed distribution of cells that increased the misassignment of cells to trajectory branches. In the multifurcation cases, the accuracy was overall lowered in all four metrics compared to the bifurcation cases because of the increased complexity (Fig. 5C and D). Notably, 500 HDGs were able to represent the four-way multifurcation, while 500 HVGs incorrectly exhibited a linear trajectory (Fig. 5C). Overall, HDG and HEG showed a favorable or comparable trajectory estimation compared to SeuratVst when 2000 features were used, however, they outperformed SeuratVst when 500 features were used. Moreover, only 500 HDG and HEG features achieved trajectories comparable to those obtained with 1000 and 2000 features. This demonstrated the capability of HDG and HEG for efficient and accurate estimation of cell trajectories.

### Similarity and functional analysis of selected features

**Figure 6:**
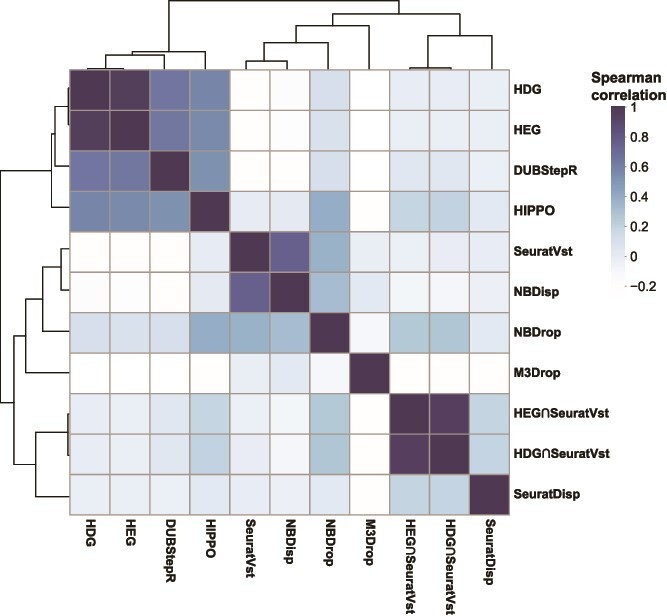
Hierarchical clustering of 11 feature selection methods based on the ranks of selected features. Spearman rank correlation was used for the similarity measure.

We performed hierarchical clustering analysis between 11 feature selection methods based on the ranks of selected features (Fig. 6). Features were selected from PBMC-sorted data. Clustering was performed after sctransform gene filtering and pheatmap R package was used to visualize the clusters.

HDG and HEG showed the highest similarity and they also formed a cluster with DUBStepR and HIPPO. SeuratVst and NBDisp, and the two hybrid methods also showed a pairwise similarity, while NBDrop, M3Drop and SeuratDisp showed a distinct feature selection. Then, we compared the functional enrichments between feature selection methods using preranked GSEA [30] and REACTOME pathways [31]. Top 20 significant pathways (adjusted *P*-value < .05) detected for each feature selection method were pooled and summarized in Figure S9.

Interestingly, eight methods other than DUBStepR, SeuratDisp and M3Drop commonly detected pathways related to development (e.g. REGULATION_OF_EXPRESSION_OF_SLITS_AND _ROBOS), proliferation (e.g. EUKARYOTIC_TRANSLATION _INITIATION) as well as immune system (e.g. NEUTROPHIL_DEGRANULATION, SIGNALING_BY_INTERLEUKINS, INFLUENZA_INFECTION) reflecting the 10 immune cell types. In particular, NEUTROPHIL _DEGRANULATION pathway was detected within top 20 ranks by all feature selection methods. Despite different orders of selected features, majority of feature selection methods exhibited some similarity in functional enrichments. Notably, the correlated group HDG, HEG, and HIPPO commonly detected multiple cell cycle, proliferation, and mitosis pathways, whereas SeuratVst, NBDisp, and M3Drop detected cell signaling pathways (e.g. GPCR_LIGAND_BINDING, CLASS_A_1 _RHODOPSIN_LIKE_RECEPTORS), characterizing respective feature selection groups.

## Discussion

Here, we benchmarked eleven unsupervised feature selection methods for scRNA-seq data analysis using two performance criteria. We demonstrated that the capability to include many ground-truth (or marker) genes did not necessarily lead to accurate cell clustering. This implied current practice that focused on gene variability only was not sufficient to address the cell distribution and ground-truth features did not make an equal contribution to clustering analysis. We found that the CVs in lowly expressed genes were orders of magnitude greater than those of highly expressed genes, ascribed to relatively high technical noise in lowly expressed genes. This means many lowly expressed genes have been being selected based on their high technical, not biological, variability. Indeed, the high-performance feature selection methods for cell clustering and data visualization, such as HDG and HEG mostly excluded lowly expressed features, while other methods selected features evenly. This was consistently observed for both simulation and real scRNA-seq data analyses and different numbers of features used. Moreover, HDG and HEG clearly separated the same cell types from different tissue origins, while SeuratVst represented them in the same cluster.

It has been assumed that lowly expressed genes can also contribute to distinguishing cell types. For example, SeuratVst weighed lowly and highly expressed genes equally in its mean–variance regression model. This regression model reflected the relatively high noise levels in lowly expressed genes by standardizing gene expression using estimates of mean and variance. However, variability of such standardized gene expression may not provide a firm ground for selecting biologically variable features, because accurate estimation of mean and variance of lowly expressed genes is extremely difficult. Indeed, the estimates of mean and variance parameters of scRNA-seq data were highly dispersed for lowly expressed genes [32]. Although lowly expressed and biologically variable genes can contribute to cell variability, unsupervised methods to select such genes risk incorporating many lowly expressed but only technically variable genes. Thus, we would recommend preselecting potentially important genes instead of including lowly expressed features.

Our results indicated that HDG, HEG, HIPPO, and NBDrop that mostly used highly expressed features were able to provide sufficient resolution to distinguish subtle differences between cell types and tissues. We further tested the effects of feature selection on trajectory analysis using a recent benchmark, and HDG and HEG also compared favorably with SeuratVst in estimating the trajectories. Notably, with the low CVs in the selected features, HDG and HEG achieved a high accuracy in downstream analysis using only hundreds of features, enabling an efficient management of large-scale single-cell data.

In addition, HEG performed as well as HDG, and even better in our mouse example, the reason of which was not clearly known [8]. Our tests suggested that the simple highly expressed genes included a sufficient number of differentially expressed genes for distinguishing cell types accurately. Moreover, unlike simulation cases, we might expect that most highly expressed genes in real-world scRNA-seq data, if not marker genes, held some amount of information that helped distinguish the cell types. Together, highly deviated or expressed genes provided greater resolution for distinguishing cell types compared to lowly expressed genes, and treating them equally caused less accurate downstream results. Our results suggested using mean expression level of a gene as an important, if not unique, criterion for future development of unsupervised feature selection methods for scRNA-seq data.

## Materials and Methods

### Simulation of scRNA-seq data

We simulated scRNA-seq read counts based on the negative binomial (NB) model using the splatter package [16]. We simulated 5000 cells for eight cell types and 3000 cells for five cell types. Both datasets included 10 000 simulated genes. The parameters for the eight and five cell type data were estimated from the Alzheimer’s disease [33] and lung cancer [34] single-cell data, respectively. We used “de.prob” parameter to control the proportion of DEGs in each cluster. We used de.prob = 0.125 and 0.06–0.08 for five and eight cell type data, respectively. We used equal numbers of up and downregulated genes (de.downProb = 0.5). To control the sparsity, we controlled the depth of the count data because denser data exhibit deeper depth (lib.loc parameter). We set lib.loc = 9.11 and 6.91 for 70% and 90% sparsity, respectively. The three real scRNA-seq datasets that we analyzed had a sparsity (overall zero rate) between 86% and 96% after gene filtering (Table S2); thus, 90% sparsity was used for simulation test. Some earlier scRNA-seq technologies exhibited higher depth and lower sparsity compared to 10X Genomics technology and more strict gene filtering can also yield lower sparsity; thus, 70% sparsity was also tested. Detailed information of the simulated data is provided in Table S1.

Feature selection methods

- SeuratVst and SeuratDisp [4] use an empirical approach and are implemented in Seurat. SeuratVst (default method) fits a LOESS regression model [35] between logarithmic mean and variance of genes (${v}_i$) and standardizes the read count data (${x}_{ij}$) as follows:
  $$ {Z}_{ij}=\frac{x_{ij}-{\overline{X}}_i}{\hat{v_i}} $$
  where $\hat{v_i}$ is the regressed estimate of standard deviation for gene *i*. After clipping the maximum (square root of cell count) of the normalized value, SeuratVst ranks genes based on the variance of the Z-values across all cells.
- SeuratDisp considers the genes with large expression dispersion as variable features. It uses the variance to mean ratio (${s}_i^2/\sqrt{X_i}$) for normalized count data to select features. Seurat also provides the mvp method, which is excluded from our experiments because it uses data-dependent thresholds for mean and dispersion of genes, posing a difficulty in determining the number of features.
- HIPPO [5, 15] uses a Poisson null model for gene counts and assumes the genes with excessive zeros have substantial biological variability across cells. It uses a normal approximation to the zero proportion to select genes with excessive zeros.
- NBDrop and NBDisp [6] use NB model, and estimate mean (${\hat{\mu}}_{ij}$) and dispersion (${\hat{\gamma}}_i$) parameters for each gene *i*. NBDisp selects genes with a relatively high dispersion which is estimated from the residuals of a linear regression model,
  $$ \log \left({\gamma}_i\right)={\beta}_0+{\beta}_1\log \left({\hat{\mu}}_{ij}\right) $$
  NBDrop selects genes with a high dropout rate. The expected dropout for gene *i* is calculated as
  $$ E\left({D}_i\right)=\sum_jP\left({Y}_{ij}=0\right),\quad P\left({Y}_{ij}=0\right)={\left(1+\frac{{\hat{\mu}}_{ij}}{{\hat{\gamma}}_i}\right)}^{-{\hat{\gamma}}_i}.$$
  The *p*-value for the observed dropout for each gene is calculated using binomial distribution.
- M3Drop [6] fits the Michaelis–Menten kinetics model to the relationship between mean expression (*S*) and dropout rate (*P*), ${P}_{dropout}=1-\frac{S}{K_M+S}$. For the global parameter ${K}_M$, *t*-test is performed for the hypothesis that gene-specific parameter ${K}_i$ is equal to ${K}_M$ by estimating the error of ${K}_i$.
- HDG [8] method uses the multinomial null model to select features. This method assumes genes with constant expression across cells that fit the null model are less informative. The goodness of fit of the null model for each gene *i* can be approximately quantified by comparing the log-likelihoods between saturated and fitted models using the binomial deviance
  $$ {D}_i=2\sum_j\left[{y}_{ij}\mathit{\log}\frac{y_{ij}}{n_j\hat{\pi_i}}+\left({n}_j-{y}_{ij}\right)\log \frac{\left({n}_j-{y}_{ij}\right)}{n_j\left(1-\hat{\pi_i}\right)}\right], $$
  where ${y}_{ij}$ is the observed UMI counts for cell *j*, ${n}_j$ is the total UMI in cell *j* and $\hat{\pi_i}$ is estimated relative abundance of gene *i*. HDG selects genes with high binomial deviance values. The scry R package is used to calculate the deviance of count data. HEG selects features with high mean expression (cpm) values. The cpm values were obtained by using edgeR package [36].
- Two combination methods: HDG$\mathbf{\cap}$SeuratVst and HEG$\mathbf{\cap}$SeuratVst. For HDG$\mathbf{\cap}$SeuratVst, genes are sorted by the respective methods HDG and SeuratVst and their interactions are used. HEG$\mathbf{\cap}$SeuratVst is similarly defined.
- DUBStepR [7] prioritizes genes that represent coherent expression variation First, it constructs a gene–gene correlation (GGC) matrix using a cutoff based on the correlation range z scores. Then, it identifies a subset of seed features through stepwise regression performed on the GGC matrix. Finally, the seed subsets are expanded by adding correlated features until the feature set reaches an optimal size determined by the density index (DI) metric. DUBStepR uses a strong gene filtering (min.cell = 5%) for a reliable estimation of gene correlations.

### Normalization

We tested feature selection methods for two widely used normalization methods for scRNA-seq data: sctransform (*ver*. 2) [32] and log-normalization. Both normalized data were obtained by using SeuratVst function (with SeuratVst.flavor = ‘v2’) in sctransform package and NormalizeData function in Seurat package, respectively. Sctransform used NB count model and estimated mean and variance of each count using regularized regression model,


$$ \mathit{\log}\left(E\left({x}_i\right)\right)={\beta}_{0_i}+{\beta}_{1_i}{\mathit{\log}}_{10}(m) $$

where ${x}_i$ is the observed UMI count for gene *i* and *m* is the total count of a cell. Then, the estimated mean and variance are used to standardize each count as follows:


$$ {z}_{ij}=\frac{x_{ij}-{\mu}_{ij}}{\sigma_{ij}} $$

$$ {\mu}_{ij}=\mathit{\exp}\left({\beta}_{0_i}+{\beta}_{1_i}{\mathit{\log}}_{10}{m}_j\right),{\sigma}_{ij}=\sqrt{\mu_{ij}+\frac{\mu_{ij}^2}{\theta_i}} $$

Thereby, the normalized counts exhibit the same “stabilized” variances. Recently, the second version of sctransform was published [14]. This version removed genes with very low expression ($\mu <0.001$) and underdispersed expression (${\sigma}^2\le \mu$) which improved downstream analysis.

### Clustering analysis for simulated scRNA-seq data

We performed the graph-based clustering procedure in Seurat package (Louvain algorithm). It first calculates *k*-nearest neighbors (KNN) and constructs a shared nearest neighbors (SNN) modularity using the default parameters. The KNN was constructed using top 20 principal components; thus, the Jaccard similarity was easily computed when searching for neighboring cells. The resolution parameter of the modularity optimizer sets the granularity of downstream analysis, determining the number of clusters. We used a range of resolution parameter between 0.01 and 1.2, so that the clustering output yields the true number of cell types. Seurat clustering algorithm allows us to control the percentage of shared neighbors to be kept in KNN and SNN by setting input parameters. We used default cutoff for the Jaccard index.

### Evaluation of clustering performance

ARI measures the overlap of two sets of labels. ARI ranges between 0 and 1, which indicate the random cell labeling and the perfect match, respectively. The ARI was calculated between known cell type labels and the cluster labels. ARI was calculated using the mclust R package [37]. ASW measures the extent of separation between clusters. It ranges between −1 and 1, which indicate strong misclassification and perfect separation of clusters, respectively. ASW was implemented using the aricode R package [38].

### PBMC and Tabula Muris consortium scRNA-seq data analysis

For PBMC-sorted data, the Louvain clustering algorithm with the same modularity parameters and range of resolution as the simulation case were applied. We downloaded the endothelial cell data for Tabula Muris consortium from Figshare (https://figshare.com/articles/EC_TSNE_Robj/12170358) [19]. The processed data include 21 392 genes and 3961 cells from 12 organs. We used 3130 cells from four organs (brain, fat, heart, and lung) that included 5% or larger number of cells and filtered 4943 genes using sctransform package. In total, scRNA-seq data for 3130 endothelial cells and 16 449 genes were analyzed.

### Cell type annotation with singleR

Both inputs for singleR, immune cell scRNA-seq data (test) and FACS bulk RNA-seq data (reference) were downloaded from github (https://github.com/krasnowlab/HLCA/). The FACS bulk RNA-seq data were processed as described in github; the cells for three types (Effector Memory CD45RAp CD8 T cell, More Mature NK cell, and CD16p Dendritic cell), where a small number of genes were differentially expressed were excluded. We then filtered 1291 mitochondrial/ribosomal genes and 34 035 genes using sctransform package. We used the 12 cell types in the FACS bulk RNA-seq as the reference labels. This number was doubled by combining the two tissue origins labeled in the test data. We removed the cell types that included <1% of the cells which yielded 9 and 12 cell types without and with tissue information, respectively. In total, scRNA-seq data for 2258 immune cells and 23 357 genes were analyzed for cell type visualization.

**Key Points**

- We perform a systematic benchmark of 11 feature selection methods for scRNA-seq data analysis using two commonly used criteria.
- We show the discordance between these criteria: the widely used SeuratVst includes more marker genes than HDG and HEG methods; however, the latter perform better in clustering cells, data visualization as well as trajectory estimation. We suggest using the criterion of accurate downstream analysis.
- The coefficients of variation in lowly expressed genes are much greater than those of highly expressed genes. Indeed, high-performance methods for downstream analysis including HDG and HEG mostly exclude lowly expressed features, while other methods select features evenly.
- HDG and HEG require only hundreds of features to obtain accurate downstream results, while SeuratVst needs thousands of features. Such characteristic of HDG and HEG enables more efficient management of large-scale single-cell data.

## Supplementary Material

Supplementary_Data_final_bbae317

## Data Availability

PBMC-sorted data (FACS) [17] are available from the website https://support.10xgenomics.com/single-cell-gene-expression/datasets. scRNA-seq data for lung and circulating blood samples [21] are available at github (https://github.com/krasnowlab/HLCA). Preprocessed scRNA-seq endothelial cells data from Tabula Muris Consortium samples are available from Figshare (https://figshare.com/articles/EC_TSNE_Robj/12170358). Detailed information on the datasets is described in Methods and Supplementary Tables 1–3). Simulation data for clustering analysis and the source codes for the analyses are available at Zenodo (https://doi.org/10.5281/zenodo.10017609). Simulation data for trajectory inference [27] are downloadable from https://zenodo.org/records/1443566.

## Terms and abbreviations

SeuratVst: the default feature selection method in Seurat package

SeuratDisp: dispersion-based feature selection method in Seurat package

HDG: high deviation genes

HEG: high expression genes

CV: coefficients of variation

ARI: adjusted Rand index

ASW: average silhouette width

cpm: count per million

FACS: fluorescence-activated cell sorting

UMI: unique molecular identifier

## Author Biographies

**Juok Cho** is a graduate student of the Department of Biomedical Engineering at UNIST. Her current research interest is development of single-cell data analysis methods such as normalization, feature selection, and clustering.

**Bukyung Baik** is a post-doctoral researcher (bioinformatics) in Department of Biological Sciences at UNIST. His current research interest is integration of transcriptome data such as RNA-seq and scRNA-seq and miRNA regulatory network construction in disease.

**Hai C.T. Nguyen** is a post-doctoral researcher (computer science) in Department of Biological Sciences at UNIST and has been working in transcriptome data analysis and GWAS pathway analysis. His current research interest is developing AI methods for scRNA-seq data analysis.

**Daeui Park** is a principal researcher (bioinformatics) in Korea Institute of Toxicology. His current research interest is analysis of transcriptome responses to toxicity.

**Dougu Nam** is a professor in Department of Biological Sciences (bioinformatics) at UNIST. His research interest is omics data integration and inference of miRNA and transcription networks from multiomics data.
