# Supplementary material for: Characterizing efficient feature selection for single-cell expression analysis
Source: Brief Bioinform. 2024 Jul 8;25(4):bbae317. doi: 10.1093/bib/bbae317 (PMC11229035; doi:10.1093/bib/bbae317)
Supplement: Supplementary_Data_final_bbae317 [file supplementary_data_final_bbae317.docx]

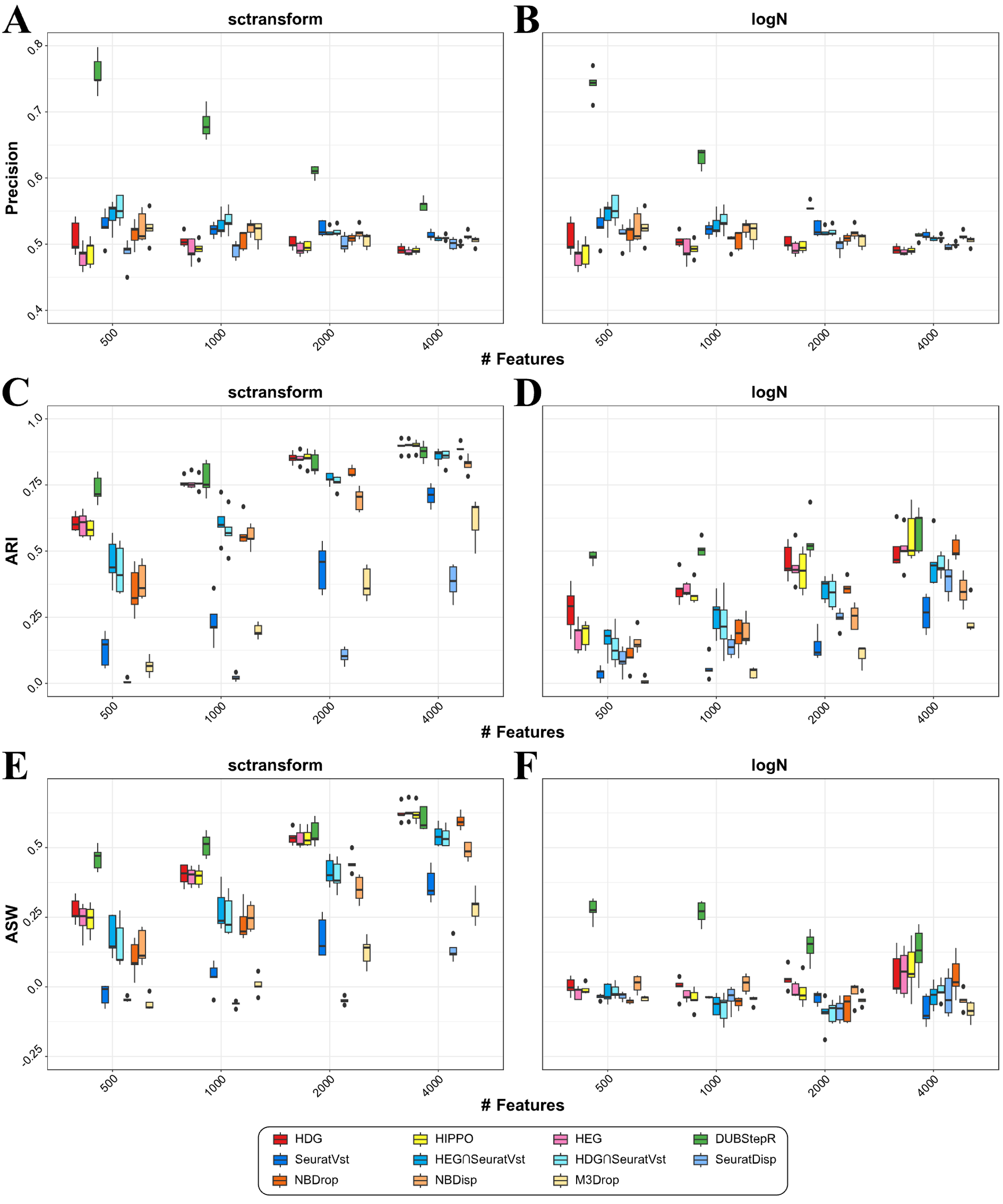


**Figure S1.** Comparison of eleven feature selection methods using two criteria: The proportion of ground-truth (differentially expressed) genes included in the features (precision) and the clustering accuracy as measured by adjusted rand index (ARI) for ground-truth cell types. The results for five simulated datasets with overall zero rates 70% after gene filtering are shown. Precision and ARI of each method are compared for different numbers of high-scoring features 500, 1000, 2000 and 4000. The precision results for **A**. sctransform and **B**. log-normalization. ARI results for **C.** sctransform and **D.** log-normalization. The average silhouette widths (ASWs) for **E**. sctransform and **F**. log-normalization are also compared

**
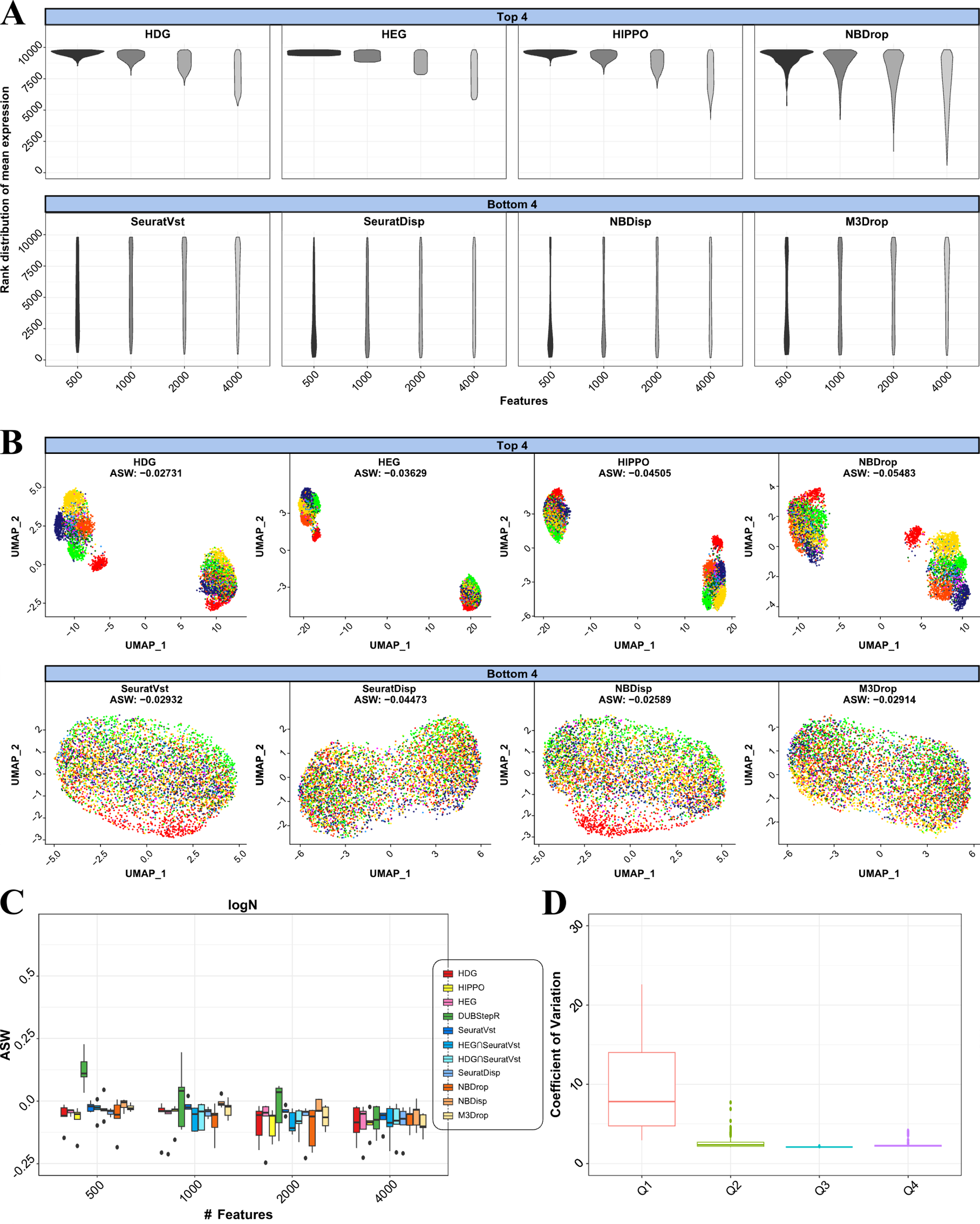
Figure S2.** The distribution of selected features and UMAP visualization are compared between feature selection methods for simulated scRNA-seq data (eight cell types). Log-normalization is used to normalize the data. **A.** Rank distribution of gene expression (mean cpm) for selected features and **B.** UMAP visualization (500 features) are compared between top four and bottom four methods in ARI. The colors in **B.** represent eight simulated cell types. **C.** The visualization of data is compared using average silhouette width (ASW) between eleven feature selection methods for 500, 1000, 2000 and 4000 high-scoring features. **D.** Coefficient of variation is compared between four quartile groups of gene expression (mean cpm). Q1 indicates bottom 25% lowly expressed genes, and Q4, top 25% highly expressed genes.


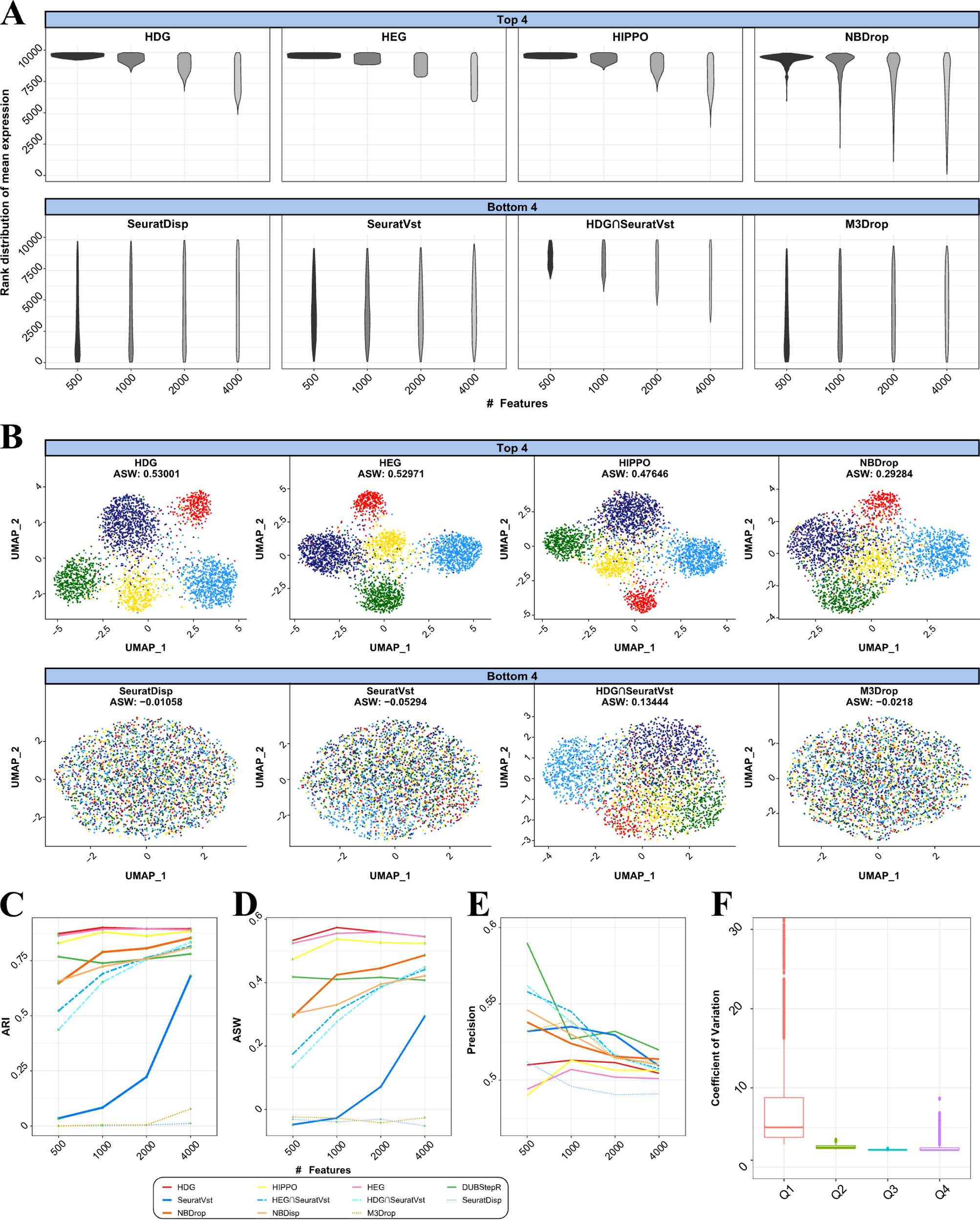


**Figure S3.** The distribution of selected features and UMAP visualization are compared between feature selection methods for simulated scRNA-seq data (five cell types). Sctransform is used to normalize the data. **A.** Rank distribution of gene expression (mean cpm) for selected features and **B.** UMAP visualization (500 features) are compared between top four and bottom four methods in ARI along with corresponding average silhouette width (ASW). The colors in **B.** represent five simulated cell types. **C.** The ARI and **D.** visualization of data (ASW) are overall measured and compared between eleven feature selection methods for 500, 1000, 2000 and 4000 high-scoring features. **E.** The proportion of ground-truth genes in the selected features (precision) is compared between feature selection methods. **F.** Coefficient of variation is compared between four quartile groups of gene expression (mean cpm). Q1 indicates bottom 25% lowly expressed genes, and Q4, top 25% highly expressed genes.


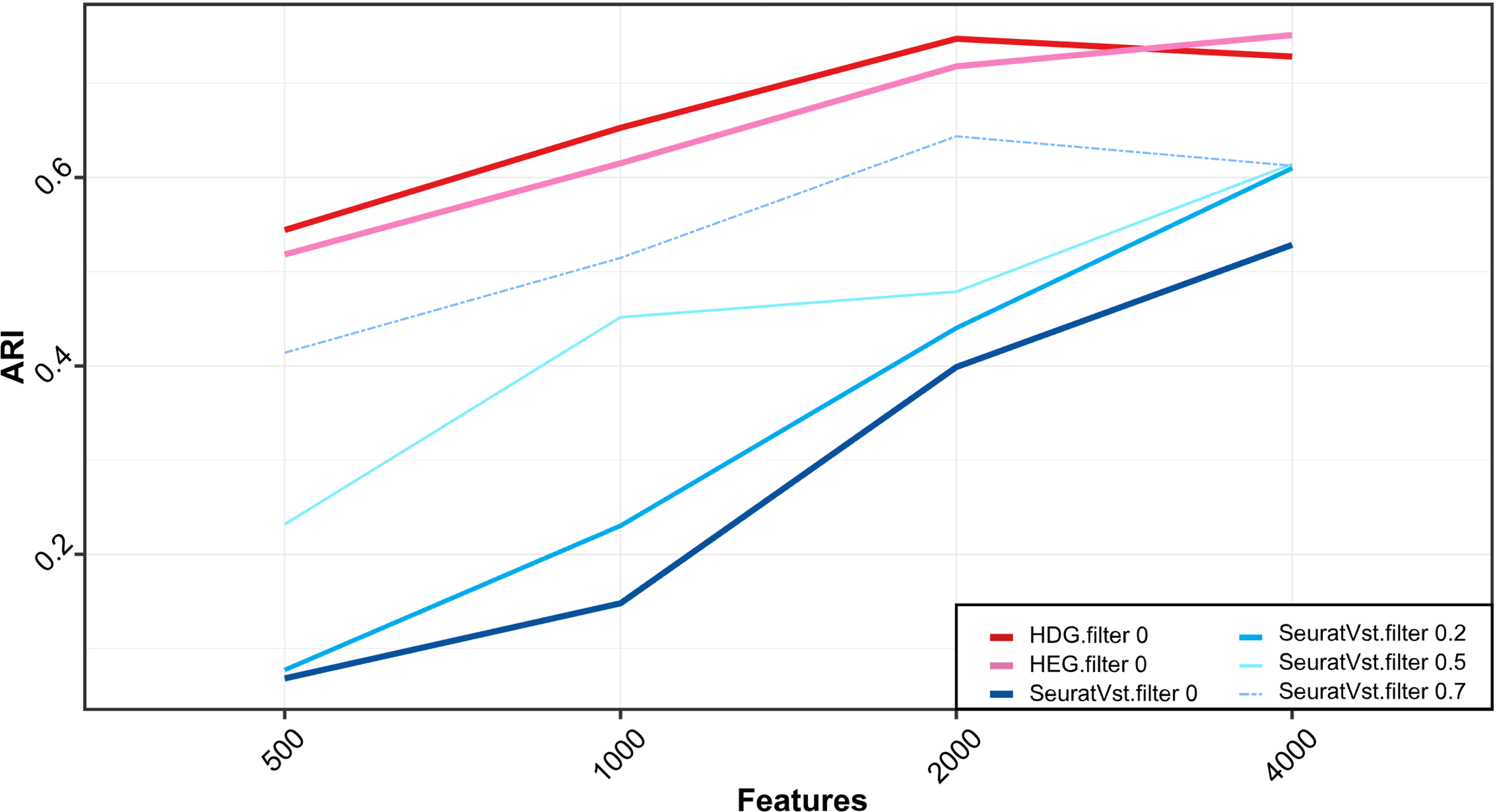


Figure S4. Comparison of ARIs with 20%, 50% and 70% lowly expressed genes filtered for SeuratVst. The simulation data with eight cell types were analyzed.


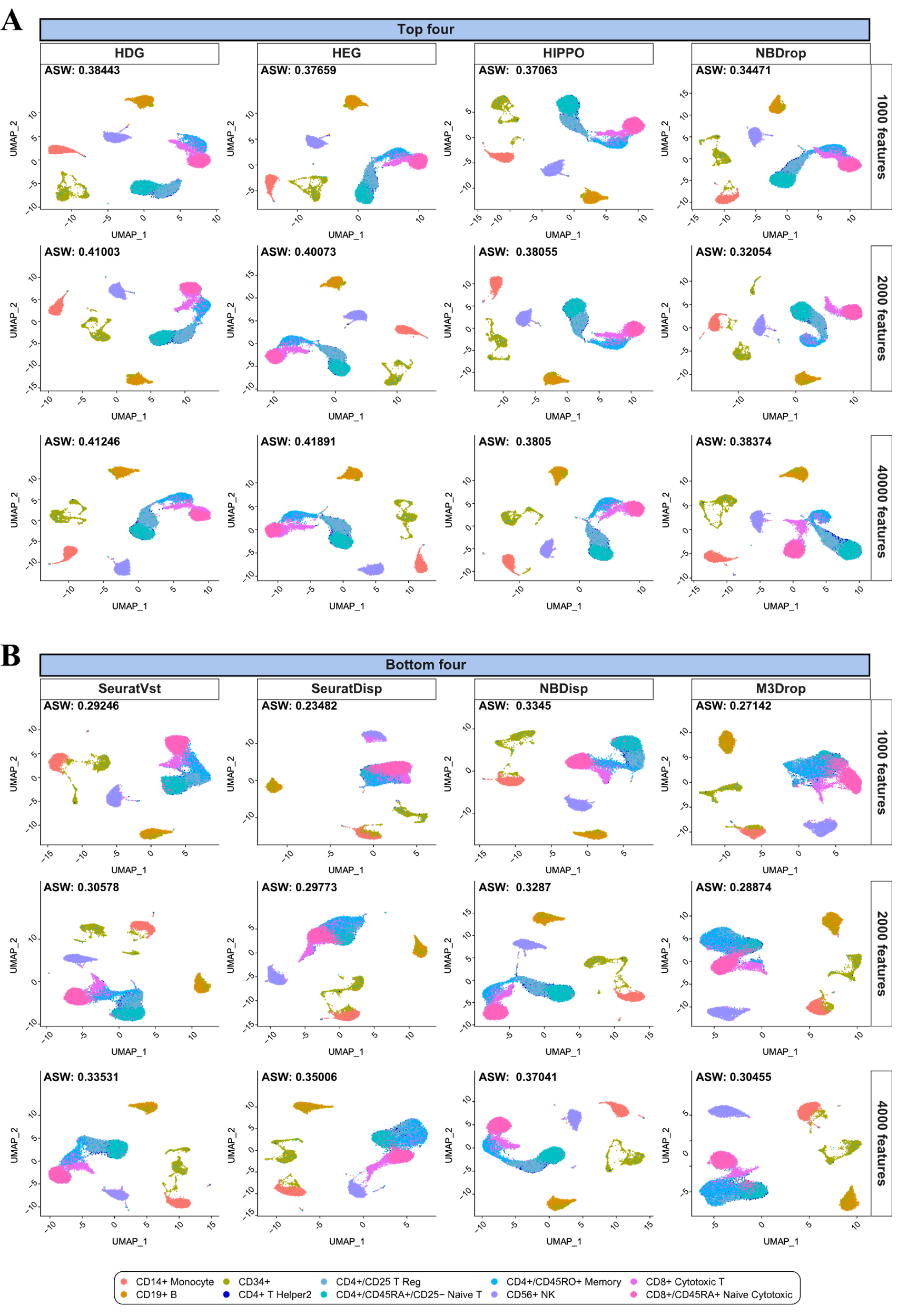


**Figure S5.** UMAP visualization is compared between feature selection methods for fluorescence-activated cell sorting (FACS) scRNA-seq data (ten PBMC cell types). Sctransform is used to normalize the data. The results for **A.** top four and **B.** bottom four feature selection methods in ARI are visualized for 1000, 2000 and 4000 high-scoring features along with corresponding average silhouette width (ASW).


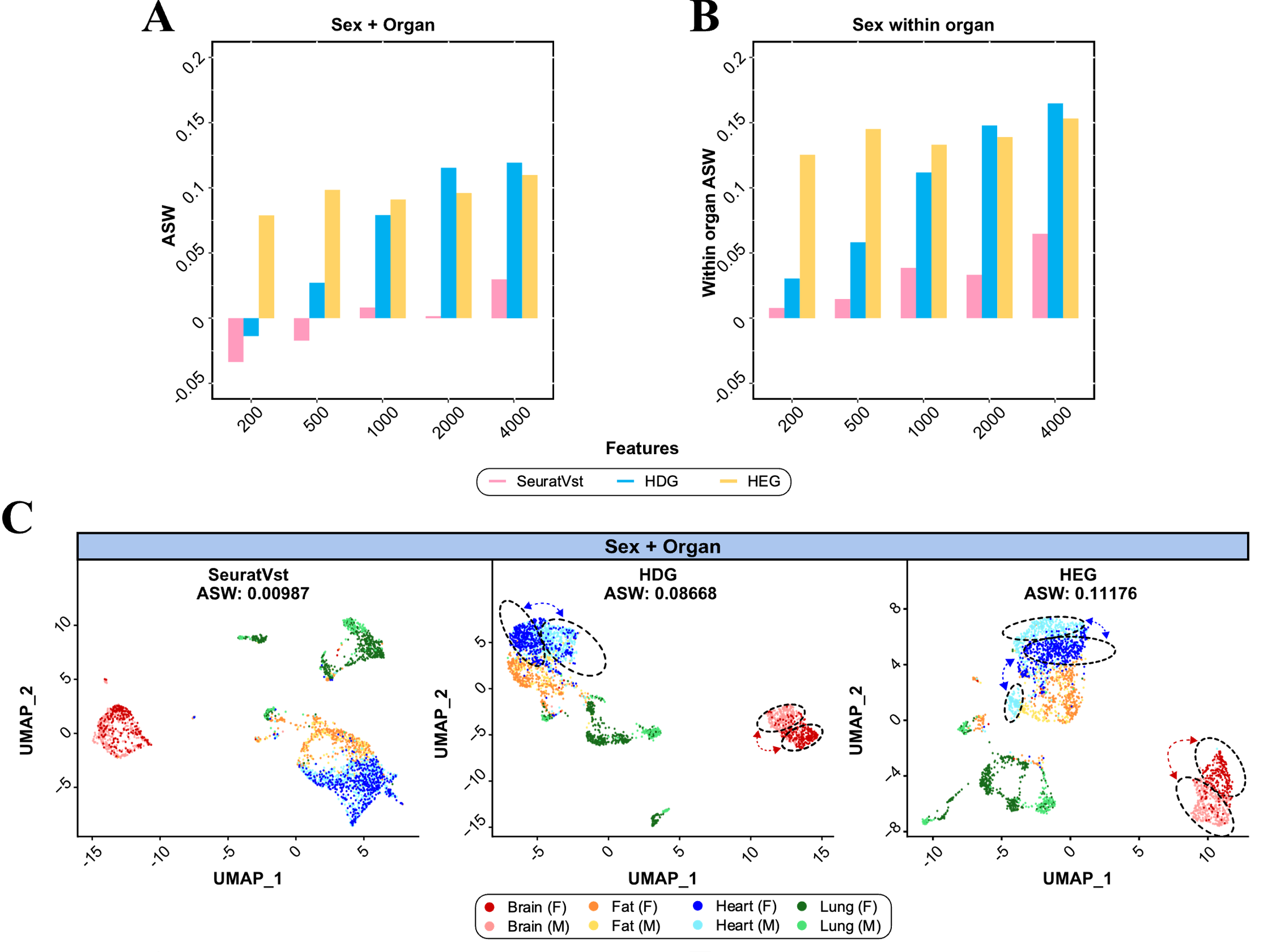


**Figure S6.** Comparisons of three feature selection methods (HDG, HEG and SeuratVst) for classifying endothelial cell scRNA-seq data. Sctransform-normalization is used. **A.** Cells were annotated with organ and sex information (eight cell categories) and the average silhouette width (ASW) is compared. **B.** ASW for sex origins within each of four organs is compared. **C.** UMAP visualization of eight combined annotations for 1000 high-scoring features. Dashed ellipses indicate brain and heart cells that are clearly separated between male and female in HDG and HEG results.


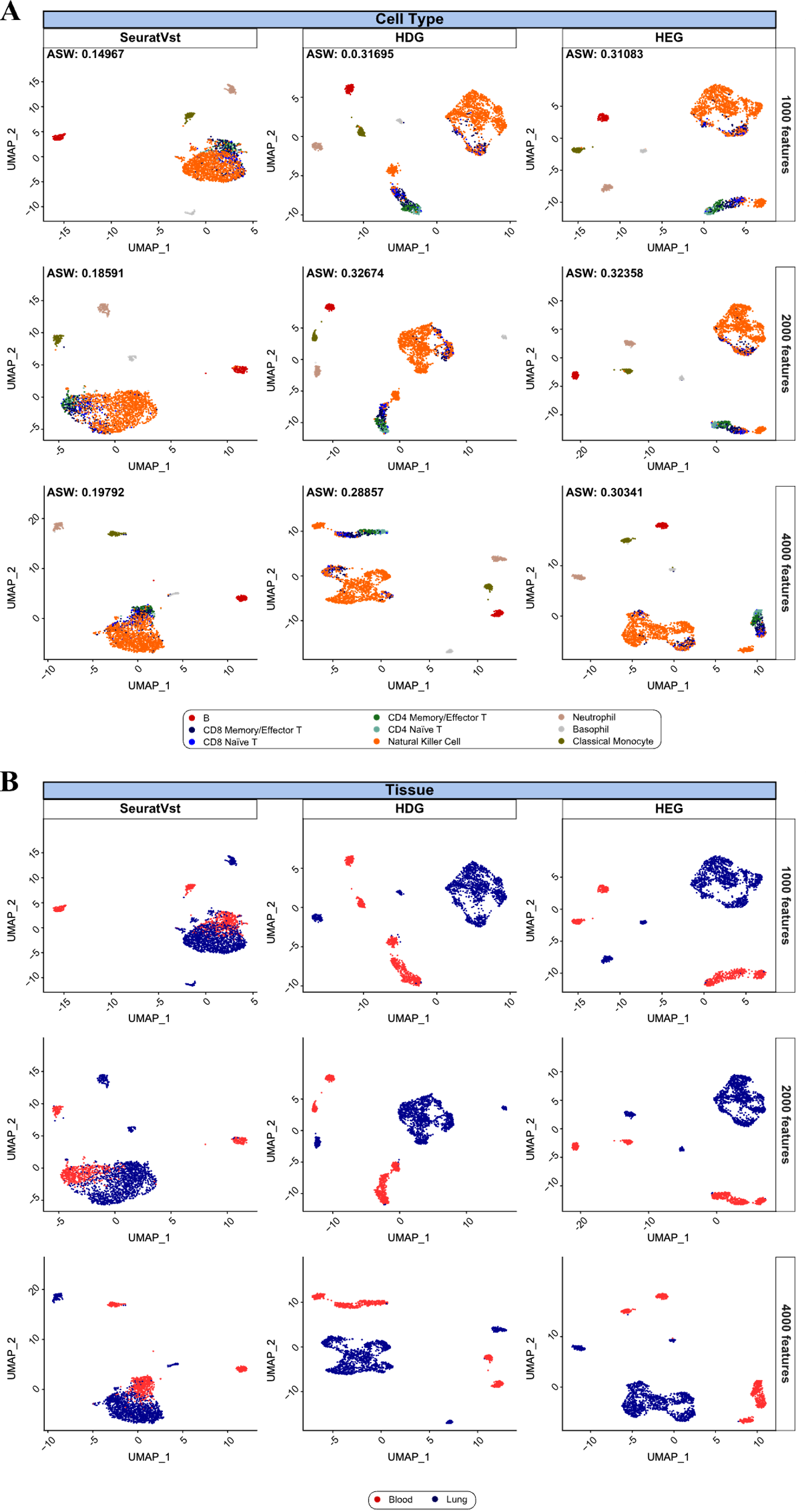


**Figure S7.** UMAP visualization of **A**. nine cell types and **B**. corresponding tissues (blood and lung) for 1000, 2000 and 4000 high-scoring features.


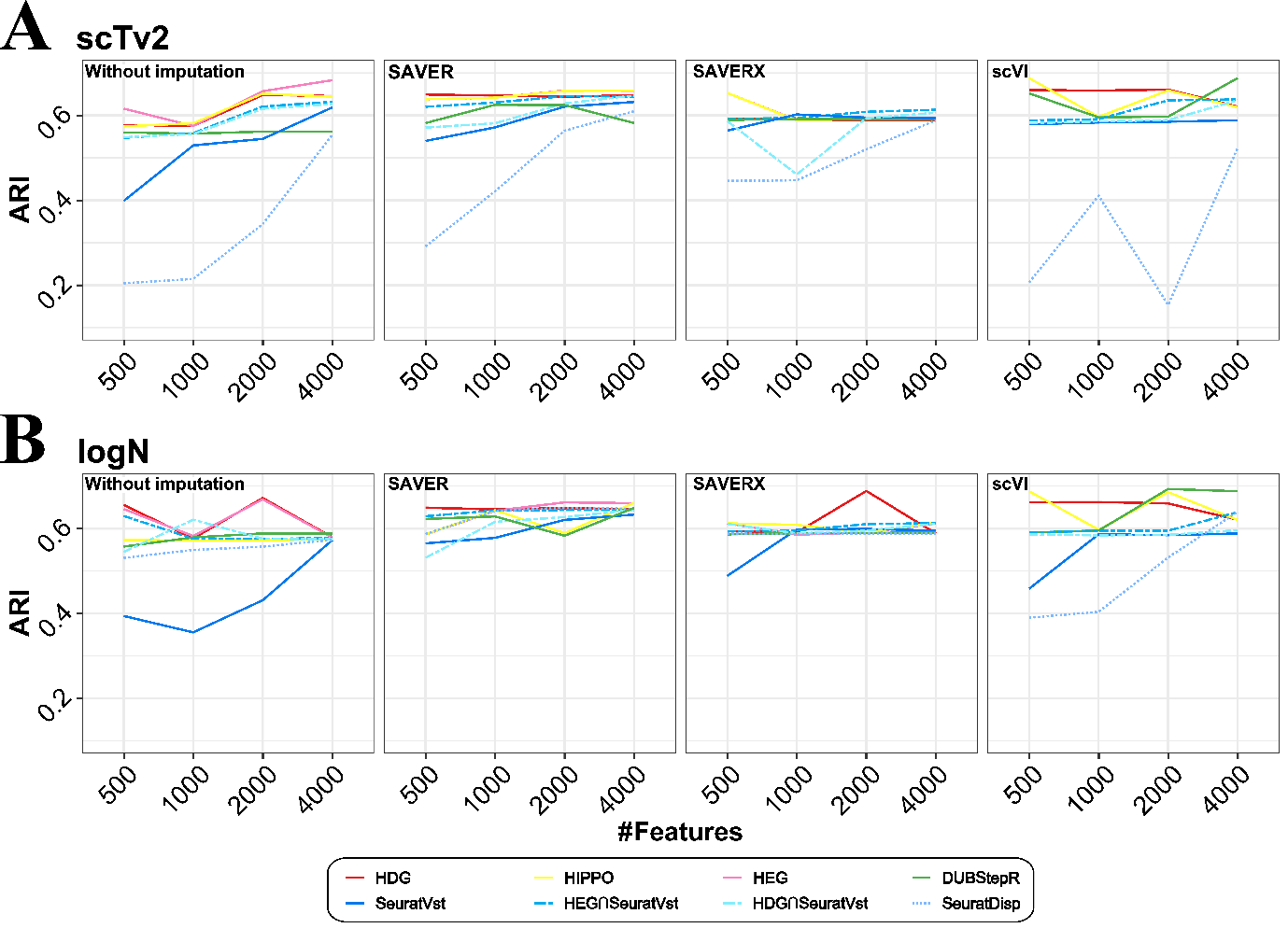


**Figure S8.** Effects of imputation on clustering analysis. Adjusted rand index (ARI) scores were compared with or without imputation for two normalization methods **A**. sctransform and **B**. log-normalization. Three imputation methods were tested (SAVER, SAVERX and scVI).


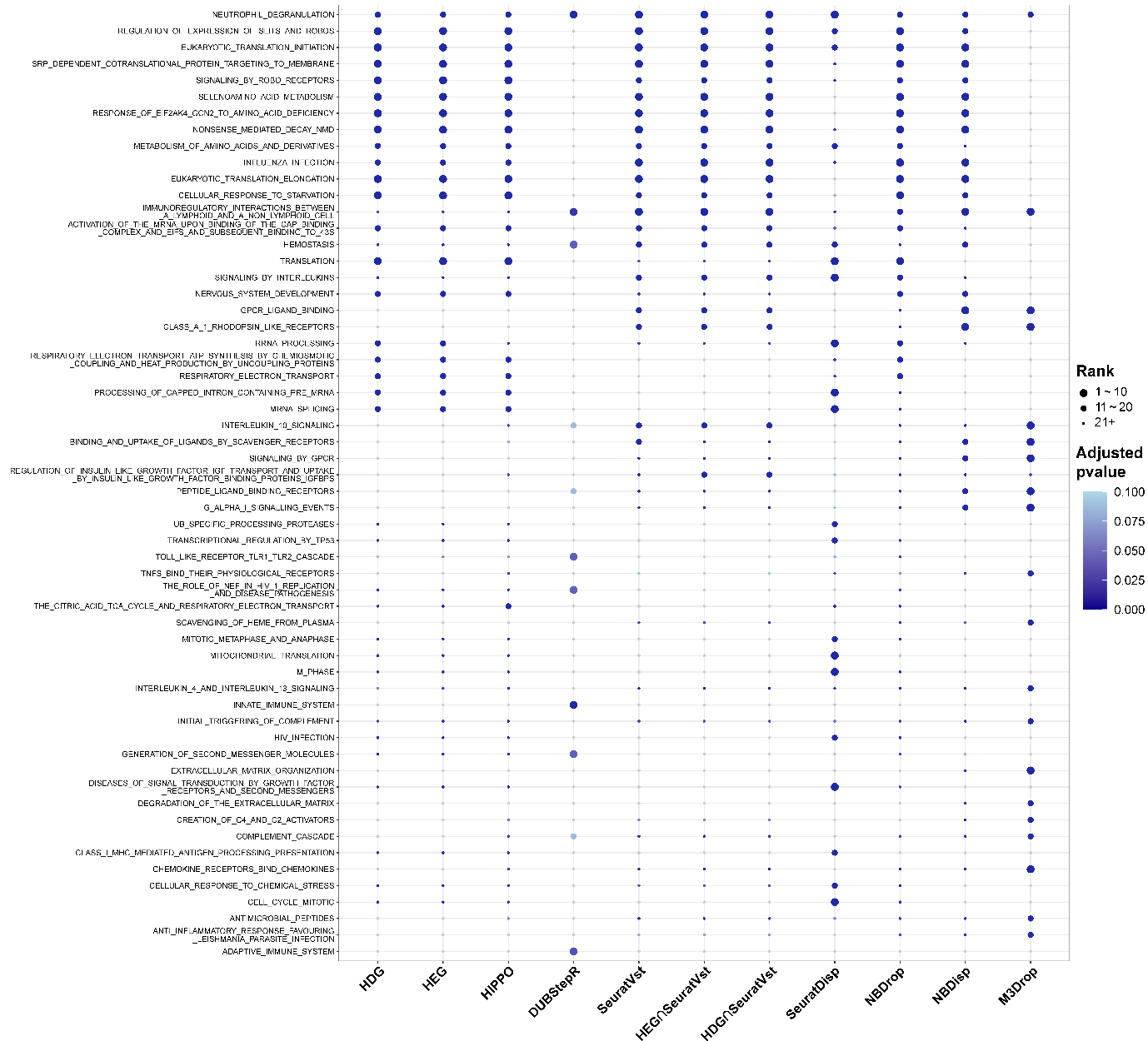


**Figure S9**. Significant pathways enriched with selected features. Preranked gene-set enrichment analysis (GSEA) was used for pathway analysis. Dot size and color indicate pathway ranks and adjusted p-value in GSEA, respectively.

**Table S1.** Simulated single-cell data used for clustering analysis in this study.

| Original data | Alzheimer  (Less sparse) | Alzheimer  (Sparse) | Lung cancer |
| --- | --- | --- | --- |
|  | Data #1 – #5 | Data #1 – #5 | Data #1 |
| #Cell types | 8 | 8 | 5 |
| #Cells | 5000 | 5000 | 3000 |
| %Cell partitions | 4:6:8:10:15:15:17:25 | 4:6:8:10:15:15:17:25 | 10:15:20:25:30 |
| #Genes | 10000 | 10000 | 10000 |
| #Total DE genes | 4931 – 4964 | 4861 – 4916 | 4941 |
| *Depth | 4.448 – 4.509 | 2.396 – 2.462 | 5.64 |
| **Sparsity | 69.0% – 69.4% | 89.0% – 89.3% | 79.1% |

*Average nonzero count after gene and cell filtering.

**Zero rate after gene and cell filtering.

**Table S2.** Single-cell data used in this study.

|  | PBMC-sorted (FACS) | Tabula Muris (FACS, endothelial cells) | Immune cells (singleR annotation) |
| --- | --- | --- | --- |
| #Cells (#after cell filtering) | 20,000 | 3,961 (3,330) | 2,367 (2,258) |
| #Genes (#after gene filtering: ribosomal, underdispersed or lowly expressed genes) | 21,952 (15,678) | 21,392 (16,449) | 58,683 (23,357) |
| #Subtypes (Annotation labels) | 10 | 4 (organ) / 8 (organ & sex) | 9 (cell type) / 12 (cell type & tissue) |
| Depth | 2.71 | 275.32 | 337.57 |
| Sparsity | 96.0% | 86.46% | 92.48% |

**Table S3**. Simulation datasets used for trajectory inference.

| Dataset | #Cells | #Genes | Trajectory |
| --- | --- | --- | --- |
| synthetic_dyntoy_bifurcating_6 | 4925 | 3870 | Bifurcation |
| synthetic_dyntoy_bifurcating_7 | 575 | 7848 | Bifurcation |
| synthetic_splatter_bifurcating_7 | 573 | 6138 | Bifurcation |
| synthetic_splatter_bifurcating_10 | 555 | 4158 | Bifurcation |
| synthetic_splatter_multifurcating_7 | 571 | 6138 | Multifurcation |
| synthetic_splatter_multifurcating_10 | 558 | 4158 | Multifurcation |
| synthetic_splatter_tree_2 | 571 | 6138 | Multifurcation |
| synthetic_splatter_tree_7 | 571 | 6138 | Multifurcation |
| synthetic_splatter_tree_10 | 558 | 4158 | Multifurcation |
